# Reactive Infectious Mucocutaneous Eruption Following Isolated Rhinovirus Infection: Clinical Presentation and Treatment Response

**DOI:** 10.7759/cureus.97457

**Published:** 2025-11-21

**Authors:** Kritin K Verma, Ethan Matthew, Sreeya Reddy, Kevin T Nguyen, Justin Raman, Floyd A Pirtle, Corley Pruneda, Michelle Tarbox

**Affiliations:** 1 Department of Dermatology, Texas Tech University Health Sciences Center, Lubbock, USA; 2 Department of Dermatology, Texas Tech University Health Sciences Center El Paso Paul L. Foster School of Medicine, El Paso, USA; 3 Department of Dermatology, University of Texas Medical Branch at Galveston, Galveston, USA

**Keywords:** blistering rash, cutaneous drug reaction, human rhinovirus, muco-cutaneous diseases, mucosal blisters, mycoplasma pneumoniae-induced rash and mucositis (mirm), reactive infectious mucocutaneous eruption (rime), severe mucositis, stevens-johnson syndrome and toxic epidermal necrolysis (sjs)

## Abstract

This case report describes a 16-year-old male who presented with a mucosal-predominant blistering eruption triggered by a rhinovirus infection, consistent with a diagnosis of reactive infectious mucocutaneous eruption (RIME). RIME is a rare condition characterized by severe mucositis and relatively mild cutaneous symptoms, in contrast to other severe cutaneous reactions like Stevens-Johnson syndrome and toxic epidermal necrolysis. RIME occurs in the setting of respiratory infections, with *Mycoplasma pneumoniae *being the most cited agent, though other viral infections, including COVID-19, have been implicated. This case is notable as it represents one of the first reported instances of RIME associated with rhinovirus alone, without the presence of other respiratory pathogens. The authors emphasize the importance of recognizing RIME as a distinct clinical entity, as reported cases of RIME have increased in recent years. Further research is needed to better characterize this condition and improve diagnostic awareness among clinicians.

## Introduction

Reactive infectious mucocutaneous eruption (RIME) is defined as a mucosal-predominant blistering eruption commonly caused by bacterial or viral respiratory infections and is characterized by severe mucositis and diverse, but often sparse, skin lesions [1]. Cases present with significant mucosal involvement of two or more mucous membranes, with minor cutaneous involvement [2]. RIME includes *Mycoplasma pneumoniae*-induced rash and mucositis (MIRM), a unique combination of erosive mucositis and rather modest cutaneous symptoms [3]. RIME differs from other severe cutaneous reactions, such as Stevens-Johnson syndrome (SJS), toxic epidermal necrolysis (TEN), and erythema multiforme major in terms of genesis, illness course, and therapy [1,3]. Recent investigations have broadened our understanding of RIME beyond its original relationship with *Mycoplasma pneumoniae*. A comprehensive narrative review revealed a growing number of viral triggers, including adenovirus, Epstein-Barr virus, influenza B, enterovirus, and SARS-CoV-2, but evidence-based therapy recommendations remain few [4]. The illness primarily affects juvenile patients and young adults, with a significant male predominance (66%), and recovery usually occurs within 7-21 days [4].

RIME differs from SJS and TEN by having prominent mucositis affecting ≥2 mucous membranes, minimal cutaneous involvement (<10% body surface area), absence of widespread epidermal necrosis, and an excellent prognosis with rare mortality [2,4]. While one prior case report described RIME in conjunction with concurrent SARS-CoV-2 and rhinovirus infection, with SJS/TEN-like characteristics [2], this is the first recorded case of RIME caused by rhinovirus as an isolated pathogen.

## Case presentation

**Figure 1:**
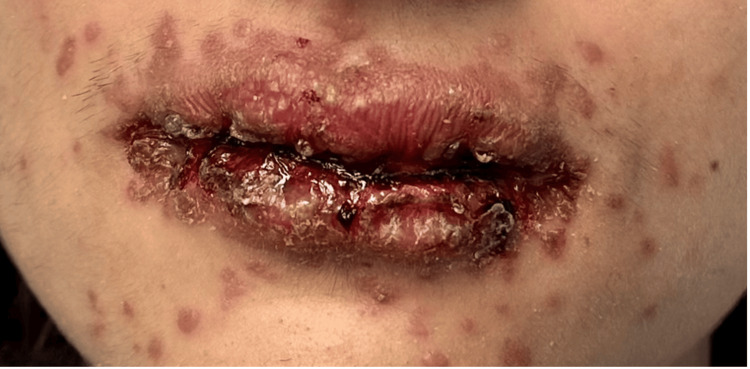
Mucocutaneous involvement of Reactive Infectious Mucocutaneous Eruption. Severe mucositis with hemorrhagic and crusted erosions of the lips, accompanied by scattered erythematous papules and targetoid lesions on the perioral skin.

**Figure 2:**
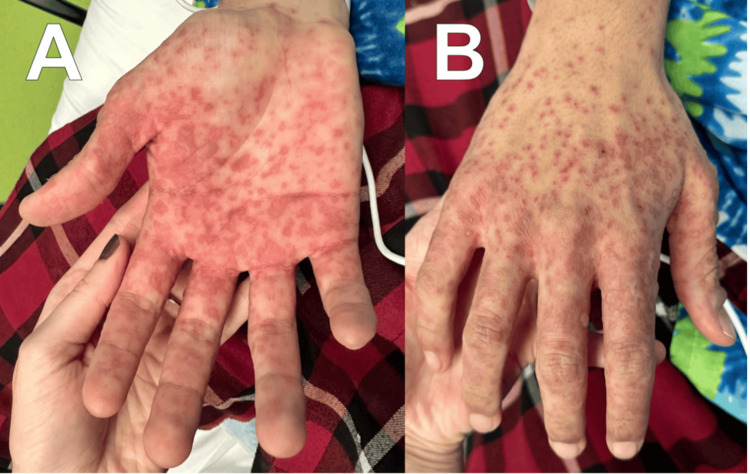
Cutaneous involvement of reactive infectious mucocutaneous eruption (RIME). Multiple erythematous macules and papules coalescing into patches on the palm (A) and dorsal hand (B), with a predilection for acral surfaces.

**Figure 3:**
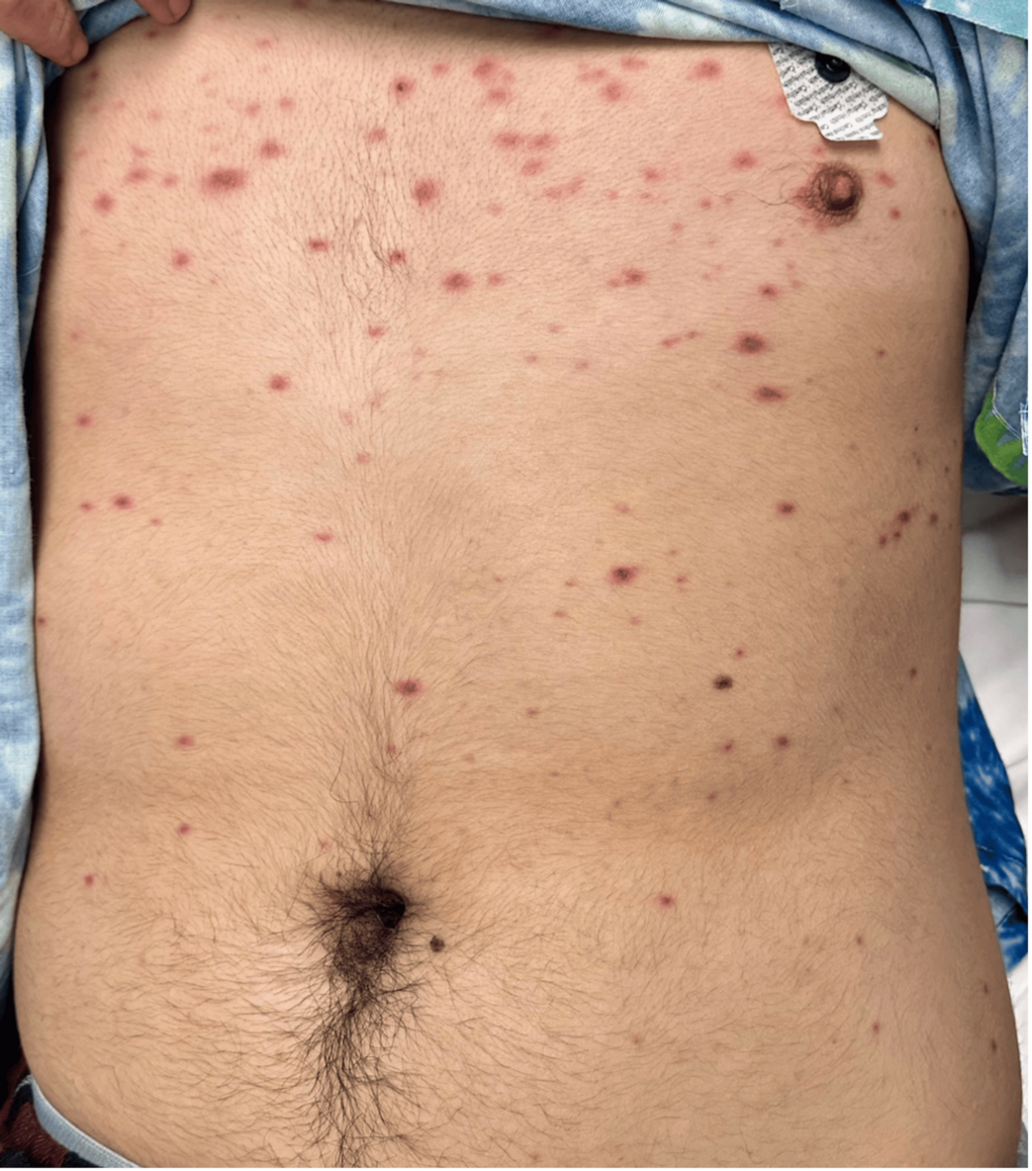
Truncal involvement of reactive infectious mucocutaneous eruption (RIME). Numerous discrete and coalescing erythematous macules and papules scattered over the abdomen, some with targetoid morphology.

A 16-year-old male was admitted to a tertiary care facility with suspicion of SJS/TEN. He started taking lamotrigine three weeks before his admission and terminated it six days prior to the onset of rash. Presentation of the rash began as erythematous macules and papules on the acral surface of the hands with eventual spread to the proximal extremities, trunk, and groin, with the involvement of the penis. Involvement of mucosal surfaces began the day after the initial presentation of the rash as small erythematous macules and progressed to vesicles and bullae of the oral mucosa and lips. The onset of fever, sore throat with accompanying cough, pain on urination, and blurred vision occurring five days after the initial presentation of the rash raised appropriate concern of worsening mucosal involvement and necessitated an emergency department visit and subsequent transfer for evaluation. Presentation of the rash on admission, as seen in Figure 1, included erythematous macules and papules of the face with hemorrhagic crusting and erosions of the lips at sites of previous vesicles and bullae. Erythematous papules and vesicles on the hands and trunk are depicted in Figures 2A, 2B, and Figure 3. Though not depicted with imagery, the patient presented with matted discharge from the bilateral eyelids and dusky macules on the penis with erythema on the urethral meatus. 

All lab values on complete blood count (CBC) were within normal limits except for a noted elevated monocyte count of 1.49 K/µL (normal: ~0.2-0.8 K/µL). The patient tested positive for rhinovirus, which was thought to be the etiology of RIME in this patient. Herpes Simplex Virus 1 (HSV-1) IgG was positive, indicating previous exposure with a low suspicion of concurrent erythema multiforme. Mycoplasma IgG and IgM tests were negative.

On the second day of hospitalization, dermatology was consulted and determined that the clinical suspicion for SJS/TEN was low, and a diagnosis of RIME secondary to rhinovirus infection was made. The patient was started on topical triamcinolone ointment 0.5% twice daily for lesions of the mouth and skin, and the patient noted improvement in pain in the oral mucosa on the second day of admission. Ophthalmology was consulted for blurred vision and noted no damage to the cornea or conjunctiva. The patient’s pain during urination improved with treatment with triamcinolone. After noticeable improvement in the mucus membrane and cutaneous lesions, the patient was discharged on the fifth day of admission with instructions for twice daily topical triamcinolone ointment 0.5% and vaseline for active lesions. Unfortunately, the patient was lost to follow-up and did not attend his scheduled appointment.

## Discussion

The pathophysiology of RIME is not well elucidated; however, it varies from the cytotoxic T-lymphocyte-mediated processes driving SJS/TEN [1,2]. RIME represents a unifying diagnosis to encompass mucocutaneous eruptions in children thought to be secondary to infectious etiologies, most notably *Mycoplasma pneumoniae *[1]. Before being reclassified to RIME, mucocutaneous eruptions secondary to *Mycoplasma pneumoniae *were termed MIRM [5]. Increased documentation of RIME in recent years has provided clarity on known causes, and the reported cases have led to increased guidance on treatment modalities [4,6]. Although MIRM has previously been associated with rhinovirus infections, recent literature has not yet documented active rhinovirus infection as a cause of RIME, and in the cited report, the eruption was described as SJS-like, without clarification on whether viral testing was performed at the time of rash onset or afterward [2,4].

The differential diagnosis of mucocutaneous blistering syndromes necessitates meticulous clinicopathological correlation [4]. In this case, RIME was preferred over SJS/TEN due to the absence of targetoid lesions, minor cutaneous detachment (<10% body surface area (BSA)), lack of extensive epidermal necrosis, and quick response to topical corticosteroids without systemic immunosuppression. The patient's lack of drug exposure within 8 weeks of symptom start suggested an infectious rather than drug-induced etiology.

Most commonly, RIME presents with mucous membrane involvement and minimal skin involvement [1]. The presentation of this patient differed in that cutaneous lesions initially appeared on the acral surfaces of the hands, subsequently spreading to the trunk, and involving nearly all mucocutaneous sites, including the oral cavity and genitalia. The patient also reported pruritus of the eyes. This patient did not display widespread skin lesions, targetoid-like lesions common in erythema multiforme, nor cutaneous necrosis or sloughing common in SJS/TEN. The patient's positive rhinovirus polymerase chain reaction and absence of other respiratory pathogens, including *Mycoplasma *infection, have not been reported in recent literature as an etiology of RIME [4,6]. A recent cohort study noted *Mycoplasma *as the most common pathogen present in up to 56% of initial cases of RIME. Other viral etiologies included adenovirus, Epstein-Barr virus, and SARS-CoV-2 [6].

Current RIME management lacks evidence-based guidelines, and treatment techniques are primarily empirical. Immunomodulatory treatments are the cornerstone of treatment, with systemic corticosteroids and cyclosporine A providing the greatest effect in severe instances [3,4]. Topical corticosteroids, as employed in this case, are an appropriate first-line treatment for limited illness [4,7]. High-potency topical treatments, such as triamcinolone acetonide 0.5%, are excellent anti-inflammatory drugs that alleviate mucositis pain and stimulate epithelium repair [8]. Severe or worsening RIME may necessitate systemic immunosuppression [3]. Etanercept, a tumor necrosis factor alpha (TNF-α) inhibitor, has shown efficacy in pediatric RIME instances [3]. Dosing at 0.6-0.8 mg/kg subcutaneously results in quick stabilization of mucocutaneous lesions [3]. However, etanercept's efficacy may be neutralized when combined with intravenous immunoglobulin; thus, this should be avoided [3]. Appropriate consultation teams, including ophthalmology and urology, should be consulted if the eyes or genitalia are involved [9, 10].

## Conclusions

RIME is a predominantly mucocutaneous eruption in children commonly associated with *Mycoplasma *pneumonia and other upper respiratory infections. This case represents an unusual presentation of RIME beginning on the hands with later widespread dissemination to the trunk, genitalia, and oral cavity, which was attributed to a rhinovirus infection. While we attributed the illness to rhinovirus, given the absence of other identifiable causes, RIME remains a diagnosis of exclusion. Because rhinovirus positivity alone does not prove it was the direct cause, and no follow-up data were available, conclusions about long-term outcomes should be made cautiously. This case represents an association between RIME and acute rhinovirus infection that has not been reported in the recent literature and should be on the differential for patients presenting with RIME.
